# Supplementary material for: Bulk band inversion and surface Dirac cones in LaSb and LaBi: Prediction of a new topological heterostructure
Source: Sci Rep. 2018 Oct 5;8:14867. doi: 10.1038/s41598-018-33273-6 (PMC6173707; doi:10.1038/s41598-018-33273-6)
Supplement: Supplementary file 1 — Supplementary Information [file 41598_2018_33273_MOESM1_ESM.pdf]

Supplementary Information – Bulk band inversion and surface Dirac cones in LaSb and LaBi : Prediction of a new topological heterostructure

Urmimala Dey[1,*], Monodeep Chakraborty[2], A. Taraphder[1,2,3], and Sumanta Tewari[4]

[1] *Centre for Theoretical Studies, Indian Institute of Technology, Kharagpur-721302, India*
[2] *Department of Physics, Indian Institute of Technology, Kharagpur-721302, India*
[3] *School of Basic Sciences, Indian Institute of Technology Mandi, HP 175005 India*
[4] *Department of Physics and Astronomy, Clemson University, Clemson, South Carolina 29634, USA*
[*] *urmimaladey@iitkgp.ac.in*

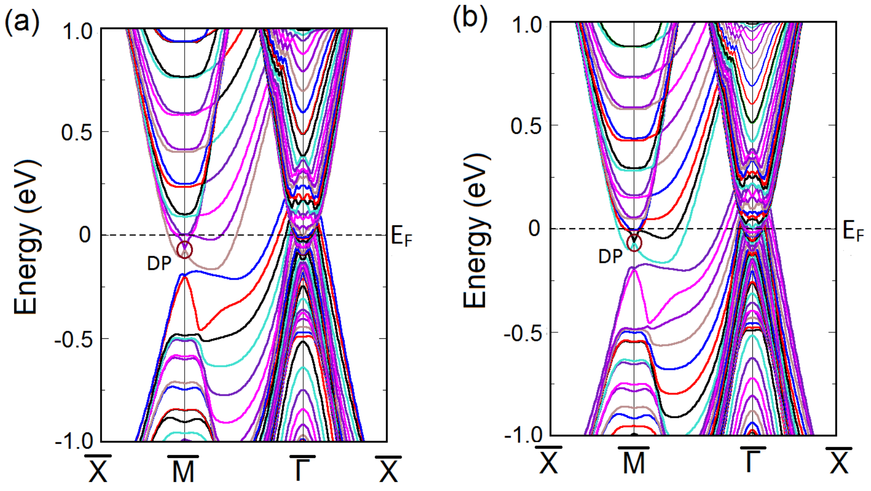

Supplementary Fig. S1 : (001) surface band structures of LaBi for two different slabs with (a) 22 layers and (b) 26 layers containing 44 and 52 atoms respectively. Our slab calculations along the high symmetry directions ($\bar{X}$-$\bar{M}$-$\bar{\Gamma}$-$\bar{X}$) for slabs of different thickness show that in all cases the Dirac cone appears at the $\bar{M}$-point, thus substantiating that the Dirac cone is a common feature independent of the choice of slab thickness.

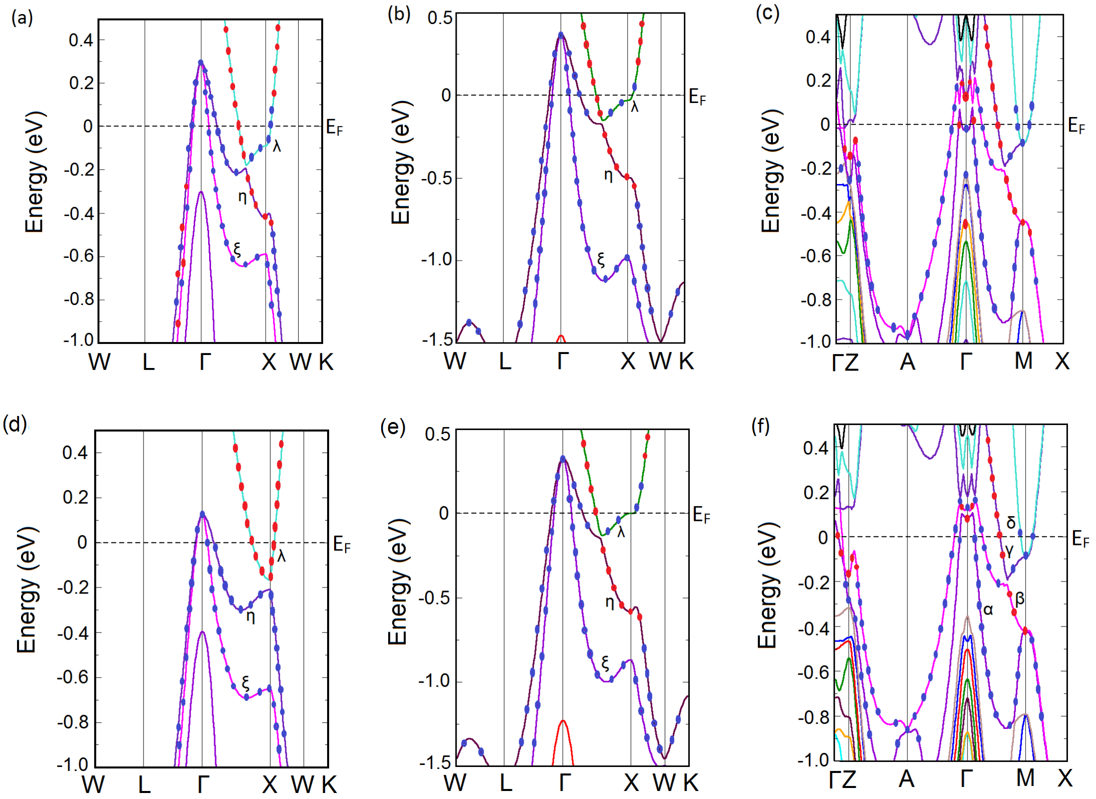

Supplementary Fig. S2 : Bulk band dispersions of LaSb, LaBi and the LaSb/LaBi multilayer respectively using (a) − (c) LDA+SO functional and (d) − (f) mBJLDA+SO functional. The red dots indicate the contribution of the +ve parity La-d orbitals and blue dots show the contribution of the -ve parity Sb/Bi-p orbitals to the band structure. For LaSb and LaBi, the conduction band and the valence band get inverted along the $\Gamma - X$ direction, whereas, in case of the multilayer, the band inversion takes place between the Γ-point and M point. In contrast to LaSb, the band inversion remains preserved in LaBi and LaSb/LaBi heterostructure when we include the mBJ potential, resulting in a non-trivial $\mathbb{Z}_2$ invariant for LaBi and LaSb-LaBi multilayer. On the other hand, the mBJLDA functional obliterates the band inversion in LaSb, resulting in a trivial $\mathbb{Z}_2$ index $\nu_0 = 0$ based on the parity criteria.
